# Different response of a native dragonfly species against a neonative invader along a latitudinal gradient

**DOI:** 10.1016/j.isci.2025.112887

**Published:** 2025-06-13

**Authors:** Koki Nagano, Masayoshi K. Hiraiwa, Naoto Ishiwaka, Francisco Sánchez-Bayo, Daisuke Hayasaka

**Affiliations:** 1 Graduate School of Agriculture, Kindai University, Nara 631-8505, Japan; 2 Faculty of Agriculture, Kindai University, Nara 631-8505, Japan; 3 School of Life and Environmental Sciences, The University of Sydney, Sydney, NSW 2006, Australia

**Keywords:** ecology, entomology

## Abstract

The poleward expansion of various organisms is accelerating due to global warming, but many aspects of these biological invasions remain unclear. One less documented aspect is the competition among native and invader species (so-called neonatives) with similar ecological niches along a latitudinal gradient. In this study, we compared the foraging behaviors across latitudes between nymphs of the native dragonfly *Orthetrum albistylum speciosum* from four different Japanese regions when confronted with its neonative counterpart, *Trithemis aurora*. The foraging intake of *O*. *albistylum speciosum* nymphs from low-latitude regions did not change in the presence of *T. aurora*, whereas native populations from higher latitudes had significantly lower intakes. Our findings imply that while no clear impacts by the neonative species are found at present, threats are expected to be more severe if the invader expands its distribution to northern regions as global warming continues unabated.

## Highlights

• Global warming drives poleward expansion of species, impacting communities
• Native dragonfly foraging declines in high-latitude areas due to neonative species
• Latitudinal variation observed: no effect on low-latitude native dragonfly populations
• Neonative species pose future threats if warming-driven expansion continues

## Graphical abstract

**Figure:**
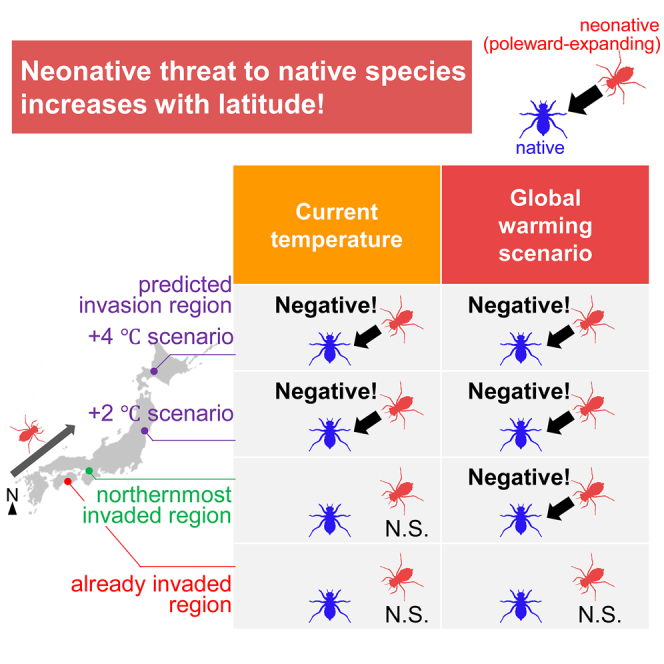

## Introduction

Global warming is progressing at an unimaginable pace^1^ and this has led to serious impacts on various organisms and ecosystems.^2^^,^^3^^,^^4^^,^^5^ Since there is little doubt that temperatures will continue to increase in the near future,^1^ predicting species responses to warming is one of the most important challenges ecologists face^5^ to understand ecosystems’ resilience and/or stability to environmental changes. Among the responses to warming, the poleward-expansion/invasion of species which originate in low latitudinal regions (i.e., horizontal movement) is known to be causing notable ecological impacts as temperatures rise.^6^^,^^7^^,^^8^^,^^9^ For example, in northern Italy, the invasion of *Aedes albopictus* Skuse (tiger mosquito) that mediates the spread of dengue fever into new areas has progressed rapidly in recent years due to global warming.^10^ Equally, the ranges of some Odonata species in Europe have shifted northwardly under increasing temperatures.^11^^,^^12^ Such biological invasions bring together organisms which would normally never be encountered and thus, could disrupt interactions in local biological communities.^13^^,^^14^ It cannot be denied that the stability of ecosystems will be compromised via invasive species that expand poleward due to warming (so-called neonative species^15^), as they will influence biotic interactions in the invaded areas.^16^ However, how the new biotic interactions develop within ecosystems under these climate driven invasions are almost never verified^17^^,^^18^ and thus, clarifying this picture is strongly required.

In assessing the invasion impacts of neonative species, which originate from low latitudes with warm environments, temperature is an important factor to consider. For instance, the diving beetle *Cybister tripunctatus lateralis* Fabricius, one of the poleward-expanding species, has been reported to have higher survival rates at elevated temperatures compared to closely related native species.^19^ In our previous study about the interspecific interaction between native (*Orthetrum albistylum speciosum* Uhler) and neonative dragonflies (*Trithemis aurora* Burmeister) we found that the threat of neonative species on the foraging intake of native dragonfly was not clearly detected under the current ambient temperature but an adverse and significant impact on food acquisition by the native species was revealed when both species were subjected to increasing temperatures.^20^ This indicates a competitive advantage of the invasive species (i.e., interference competition) under a warmer environment and the existence of a temperature dependency by the neonative species for causing ecological impacts.^20^^,^^21^ In other words, the threat posed by neonative species to the native species is not constant within a given region, but it is feared to become apparent with the progression of global warming in the future.

The invasion impacts by neonative species may also spatially vary depending on the ambient temperature of the invaded region. As it happens, biological invaders that are expanding toward high-latitudinal regions from lower ones regardless of taxonomic groups^6^^,^^7^^,^^8^^,^^9^^,^^10^^,^^11^^,^^19^^,^^22^^,^^23^^,^^24^^,^^25^ will encounter various native competitors in areas with different temperatures. Therefore, to predict changes in interspecific interactions between native and neonative species comprehensively at a broad scale, the latitudinal gradient in temperature should be taken into account. If the influence of invasive neonatives on native species varies according to ambient temperatures in each region, considering the regional temperature difference would be imperative to assess the invasion risks accurately. Since neonative species originate from warm, low-latitude regions and have an advantage under high-temperature conditions,^19^^,^^20^^,^^21^ it could be expected that their impact on native species would be stronger at lower latitudes and weaker at higher latitudes. However, this prediction might be simplistic. This is because the competitive ability against other native species may vary depending on the latitude. The fact that species diversity is higher in low-latitude regions compared to that in high-latitudinal regions^26^^,^^27^ is an additional factor that should be considered. This being the case, it may be assumed that populations of species inhabiting low-latitudinal regions with higher diversity are continuously subjected to stronger interspecies competitions than populations in higher latitudes, and this would result in the acquisition of a high competitive ability. Based on this assumption, and contrary to temperature predictions, the impact of invasive neonatives on native species is expected to be stronger at higher latitudes than weaker at lower latitudes when rising temperatures and their invasions occur in higher-latitude regions. Thus, neonative species already present in low-latitude regions could become a threat to the natives only at higher latitudes that they may invade. It is impossible to determine which prediction is closer to reality based solely on current knowledge, and thus it is necessary to verify this hypothesis using actual native and neonative populations.

**Figure 1:**
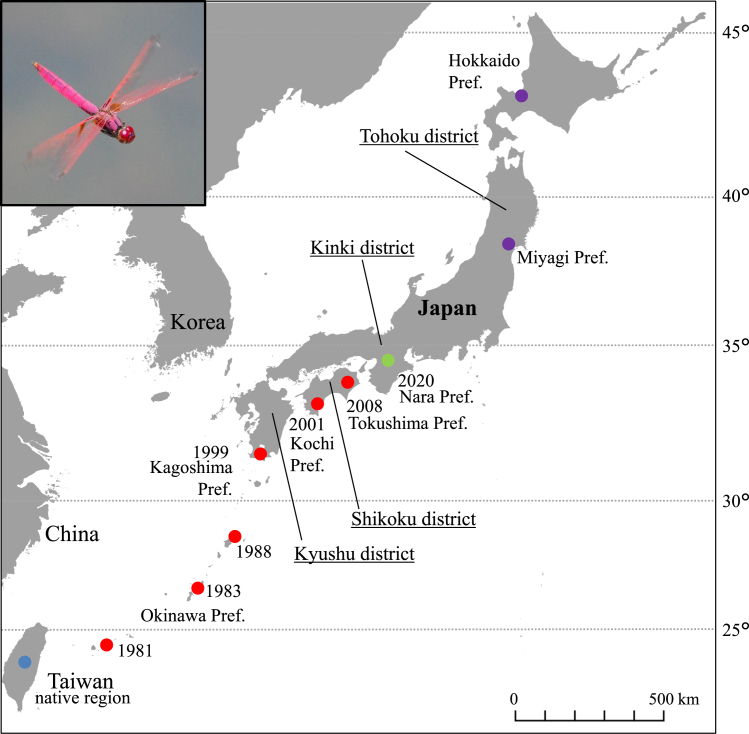
Distribution of *T. aurora*, a poleward-expanding invader dragonfly considered nowadays a neonative species in Japan This dragonfly originated in Southeast Asian regions and Taiwan, under subtropical and tropical climates, and was first detected in subtropical Ishigaki-jima Island (southwest Japan) in 1981,^32^^,^^33^ then in Okinawa Island in 1983,^33^ Amami-Oshima Island in 1988,^28^ the southern Kyushu district (Kagoshima Prefecture) in 1999,^33^ and the Shikoku district by the late 2000s^28^^,^^34^ The distribution of *T. aurora* extended to the central Kinki district (Nara Prefecture) in 2020.^20^ When the global warming scenario made by the IPCC becomes a reality,^1^ its distribution could extend to northern Japan—Miyagi Prefecture under SSP1-2.6 scenario, or Hokkaido under SSP5-8.5 scenario—by the end of this century. Symbols in this figure indicate: , native range of *T. aurora*; , its already invaded regions; , latest region of *T. aurora* detection; , regions where this species is predicted to reach by the end of 21th century due to global warming. Photo of *T. aurora* by Shunsuke Yamamoto.

To test this hypothesis, we focus here on the interspecific interactions between the invasive dragonfly *T*. *aurora* (crimson marsh glider) and its native counterpart *O*. *albistylum speciosum* (Japanese white-tailed skimmer). *Trithemis aurora* is a representative neonative species in Japan,^20^ which is native to Southeast Asia and Taiwan,^28^ but was first detected in Yaeyama and Okinawa Islands of southwest Japan (subtropical climate) around the 1980s, and then expanded its distribution to the Shikoku district (temperate climate) by late 2000s. Furthermore, this neonative species was also identified in central Kinki district (Nara Prefecture) in 2020^20^ (Figure 1). *Orthetrum albistylum speciosum* is the most common native dragonfly in Japan, belongs to the same family (Libellulidae) as *T. aurora* and both dragonflies share similar ecological requirements (e.g., habitat, prey, body size, growth period) at least at the nymphal stage.^20^ This pair of species offers the following two advantages for studying competitive interactions. First, Odonata species are good bioindicators for evaluating ecosystem stability against environmental disturbances and stresses,^29^^,^^30^^,^^31^ as they play a crucial function as intermediate predators in aquatic ecosystems during their nymphal stage or as top predators when fishes are absent.^11^^,^^18^ Second, the experimental methods for this kind of study are already established as a previous study with the same two species was conducted in Nara Prefecture, the latest region where *T. aurora* was detected in Japan.^20^

In this study, we tested whether the impact of neonative species on the foraging of native species vary with latitude.

## Results

### Effects of *T. aurora* invasion scenarios on the foraging intakes of four *O. albistylum speciosum* regional populations

**Figure 2:**
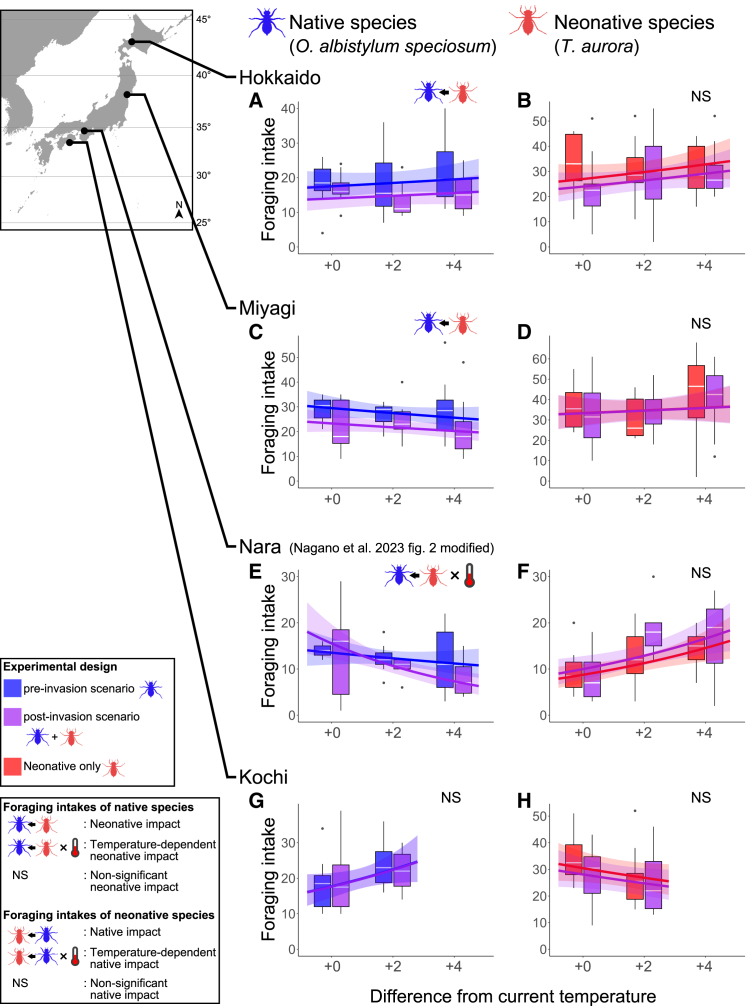
Effects of temperature and the presence of a competitor on the foraging intakes of native and neonative dragonfly nymphs (A–H) Influence of the bioinvasion scenarios of the neonative species (pre- or post-invasions) on the foraging intakes (the number of prey consumed) of nymphs of four *O. albistylum speciosum* (native) regional populations (A, Hokkaido; C, Miyagi; E, Nara; G, Kochi) at three different temperature conditions. Influence of four *O. albistylum speciosum* (native) regional populations (B, Hokkaido; D, Miyagi; F, Nara; H, Kochi) on the foraging intakes (the number of prey consumed) of nymphs of *T. aurora* (neonative) under the same temperature conditions. Fitted curves and bands for 95% confidence interval with GLMMs. The icons indicate the significant effects (*p* < 0.05); NS shows non-significant effects. The results for Nara are modified from Figure 2 of Nagano et al.^20^ See also Tables 1 and 2.

Except for the Nara population of *O. albistylum speciosum*, the foraging intake of this species in other regional populations showed no significant interactions between the temperature and *T. aurora* invasion scenarios, nor between the body size and invasion scenarios (*p* > 0.05, GLMMs). We reanalyzed the data excluding these interactions, and showed that *O. albistylum speciosum* population in Kochi (the southernmost region of our collection sites) had no significant difference in the foraging intakes for both foraging tests, i.e., pre-invasion versus post-invasion scenarios of *T. aurora* (*p* = 0.945; Figure 2G; Table 1). In contrast, the foraging intakes of populations in higher latitudes such as Miyagi and Hokkaido decreased significantly regardless of temperatures in the scenario of *T. aurora* invasion (*p* < 0.05; Figures 2A and 2C; Table 1). Interestingly, in the Nara population, located at an intermediate latitude among the study sites, there was a significant interaction between the temperature and invasion scenario of the neonative species against its foraging intakes.^20^ This pattern was intermediate between the low- (Kochi) and high-latitudinal (Miyagi and Hokkaido) results: the effect of a neonative species was small under current temperature conditions but the intakes of native dragonfly decreased under hightemperature conditions in the post-invasion scenarios (*p* = 0.021; Figure 2E; Table 1). In Hokkaido, Miyagi, and Kochi prefectures, the foraging intakes of *O. albistylum speciosum* increased with body size irrespective of *T. aurora* invasion. On the other hand, the intake of Nara population increased more significantly with body size when *T. aurora* was present, compared to the situation without the neonative species (Table 1).

**Table 1:** Effects of temperature, invasion scenario, and body size on the foraging intake of native dragonfly nymphs across four regions

Region	Variables	Estimate	S.E.	*z*	*p*	Reference
Hokkaido	Intercept	1.675	0.908	1.844	0.065	Present study
	Temperature	0.027	0.033	0.808	0.419	
	*T.a* invasion scenarios	−0.225	0.107	−2.104	**0.035**∗	
	Body size	0.209	0.097	2.151	**0.032**∗	
Miyagi	Intercept	3.581	0.748	4.789	**<0.001**∗	Present study
	Temperature	−0.035	0.028	−1.234	0.217	
	*T.a* invasion scenarios	−0.236	0.052	−4.506	**<0.001**∗	
	Body size	0.242	0.080	3.022	**0.003**∗	
Nara	Intercept	3.931	1.175	3.346	**<0.001**∗	Nagano et al.^20^
	Temperature	−0.047	0.046	−1.027	0.305	
	*T.a* invasion scenarios	2.945	1.574	1.871	0.061	
	Body size	−0.009	0.055	−0.156	0.876	
	Temperature × *T.a* invasion scenarios	−0.141	0.061	−2.318	**0.021**∗	
	Body size × *T.a* invasion scenarios	0.162	0.069	2.352	**0.019**∗	
Kochi	Intercept	−1.215	1.900	−0.639	0.523	Present study
	Temperature	0.118	0.064	1.857	0.063	
	*T.a* invasion scenarios	−0.005	0.069	−0.069	0.945	
	Body size	0.252	0.110	2.286	**0.022**∗	

Parameters of the GLMMs used for assessing the effects of temperature, *T. aurora* invasion scenarios, and body size of native species on the foraging intake (the number of prey consumed) of nymphs of *Orthetrum albistylum speciosum* in four regional populations with different latitudes (∗*p* < 0.05). Where the interactions were not significant (*p* > 0.05), we reanalyzed the data by excluding the interactions.

### Effects of four *O. albistylum speciosum* regional populations on the foraging intakes of *T. aurora*

In all regions, the foraging intakes of *T. aurora* nymphs showed no significant interactions between the temperature and presence or absence of a native species, or between the body size and presence or absence of a native species (*p* > 0.05). Thus, data excluding these interactions were reanalyzed and results indicated that, in contrast to the results for *O. albistylum speciosum*, the intakes of the neonative species did not change between the two foraging experiments regardless of a native species (i.e., different regional populations of *O. albistylum speciosum*) (*p* > 0.05). The results imply that the threat of the presence of *O. albistylum speciosum* on the foraging behavior of *T. aurora* is quite low (Figures 2B, 2D, 2F, and 2H; Table 2). Incidentally, although the foraging intake of *T. aurora* increased with rising temperatures in Nara (*p* = 0.003),^20^ this effect was not significant in other regions (*p* > 0.05) (Table 2). Furthermore, *T. aurora* intakes significantly increased with body size in regions other than Nara (*p* > 0.05) (Table 2).

**Table 2:** Effects of temperature, native species presence, and body size on the foraging intake of neonative dragonfly nymphs

Region	Variables	Estimate	S.E.	*z*	*p*	Reference
Hokkaido	Intercept	1.227	1.035	1.186	0.236	Present study
	Temperature	0.049	0.035	1.393	0.164	
	p/a of native *O.a.s*	−0.119	0.077	−1.539	0.124	
	Body size	0.320	0.110	2.898	**0.004**∗	
Miyagi	Intercept	2.391	0.970	2.464	**0.014**∗	Present study
	Temperature	0.019	0.037	0.511	0.609	
	p/a of native *O.a.s*	−0.003	0.068	−0.038	0.969	
	Body size	0.211	0.088	2.414	**0.016**∗	
Nara	Intercept	−0.739	1.379	−0.536	0.592	Nagano et al.^20^
	Temperature	0.127	0.043	2.950	**0.003∗**	
	p/a of native *O.a.s*	0.132	0.084	1.578	0.115	
	Body size	−0.089	0.068	−1.304	0.192	
Kochi	Intercept	4.364	1.280	3.409	**<0.001**∗	Present study
	Temperature	−0.063	0.040	−1.563	0.118	
	p/a of native *O.a.s*	−0.081	0.123	−0.655	0.512	
	Body size	0.309	0.108	2.859	**0.004**∗	

Parameters of the GLMMs used for assessing the effects of temperature, presence or absence (p/a) of a native species (*O*. *albistylum speciosum*), and body size of neonative species on foraging intakes (the number of prey consumed) of nymphs of a neonative species (*T. aurora*) (∗*p* < 0.05). For all regions, where the interactions were not significant (*p* > 0.05), we reanalyzed the data by excluding the interactions.

### Predation events between *T. aurora* and *O. albistylum speciosum*

In this experiment, very few *O. albistylum speciosum* nymphs in Miyagi (one individual at 27°C) and Hokkaido (one at 25°C) were preyed upon by *T. aurora*. In contrast, no *T. aurora* nymphs were preyed upon by *O. albistylum speciosum* throughout the experiments.

### Latitudinal gradient of a morphological trait of *O. albistylum speciosum*

The length of caudal appendages in *O. albistylum speciosum* collected from six regions were longer in lower latitude regions and shorter in higher latitude regions (estimate = −0.003, S.E. = 0.001, z = −4.704, *p* < 0.001, GLMM) (Figure S1). In contrast, the rearing temperatures did not have any significant effect on the length of the appendages (estimate = −0.001, S.E. = 0.001, z = −0.392, *p* = 0.695).

## Discussion

Our study revealed a new perspective about the ecological impacts or threats that poleward-expanding species have on native species, as the impacts change according to a latitudinal gradient, and their strength increase in higher latitude regions (Figure 2; Tables 1 and 2). This viewpoint has rarely been discussed until now. As we hypothesized, adverse impacts of *T. aurora* invasion on the foraging intake of nymphs of the native dragonfly *O. albistylum speciosum* clearly differed among populations at different latitudes. At the current temperature, the foraging intake of *O. albistylum speciosum* in the low-latitude regions of Kochi and Nara was not affected by the invasion of neonative *T. aurora* (Figures 2E and 2G). However, in the high-latitude regions of Miyagi and Hokkaido (Figures 2A and 2C), where *T. aurora* has not yet invaded, the foraging intake of the native dragonfly nymphs has significantly decreased (Table 1). Although the foraging intakes of *O*. *albistylum speciosum* in Kochi, the southernmost regional population of this study, did not change regardless of *T. aurora* invasion scenarios as the temperature was increased (Figure 2G), the intakes of nymphs in populations from higher latitudes (Miyagi, Hokkaido) significantly decreased in the situation after its invasion (Figures 2A and 2C). In Nara, an intermediate latitudinal region among the study sites, the impact of *T. aurora* on the foraging intake of *O. albistylum speciosum* was not constant, as it changed depending on the temperature tested, and body size appears to have influenced the outcome of their competition (Figure 2E; Table 1).

In fact, some researchers indicate that the decline of the ratio of a resource acquisition can directly lead to the decrease in the fitness of various organisms. ^35^ If so, it is envisaged that the survival rate of *O. albistylum speciosum* may decrease when encountered *T. aurora* in regions of high latitude. Also, the one-sided predation by *T. aurora* for high-latitudinal populations of *O. albistylum speciosum* can be regarded as asymmetrical intraguild predation, which may subsequently result in the displacement of native dragonfly species.^20^^,^^36^ Therefore, as the distribution of *T. aurora* expands northward due to global warming, its impacts on native species via interference competition will be stronger among those species which inhabit high-latitudinal regions and could, eventually cause the local extinction of native species. Inevitably, areas subjected to the threat of *T. aurora* (e.g., species displacement) will continue to increase as long as the global warming persists. Although both dragonfly species tested share similar ecological properties at least at the nymphal stage,^20^ it cannot be assumed that they perform exactly the same role within the ecosystem. Since Odonata species play an important role in the food web of both aquatic and terrestrial ecosystems,^30^ the species displacement caused by the neonative species in this study may lead to changes in the stability and interaction within local ecosystems.^13^^,^^29^ In contrast, there were no clear impacts from *O. albistylum speciosum* to *T. aurora* irrespective of the latitudinal gradient, and this indicates a unilateral threat from the neonative species against the native species (Figure 2; Table 2).

We propose two opposing hypotheses to explain the variation in the strength of interference competition between *O. albistylum speciosum* populations and *T. aurora*. The first hypothesis posits that invasive species, which originate from warmer regions, are more advantageous in lower latitudes among their invaded regions. The second suggests that higher latitude regions with relatively low species diversity have lower defensive ability against competitors compared to regions in lower latitudes that have higher species richness, thus making native species more susceptible to the impacts of the invader species. While both hypotheses may be valid, our foraging experiments indicated that the latter effect was more pronounced. The biotic resistance hypothesis states that ecosystems with high biodiversity have greater resilience, and so do their species when compared to ecosystems with lower biodiversity.^37^^,^^38^ The explanation for this could be that ecosystems with high biodiversity have fewer vacant niches and are more likely to contain predators and/or diseases that create a defensive attitude among the species they contain.^37^^,^^38^ In fact, the diversity of dragonfly species and other organisms tends to be higher in low-latitude regions,^34^ which supports our results. Supplementary, we compared morphological traits of *O. albistylum speciosum* by adding some new regions (Kagosima and Ibaraki prefectures) to the four regions (Kochi, Nara, Miyagi, and Hokkaido prefectures) originally tested. As a result, in each *O. albistylum speciosum* regional population, the length of the caudal appendages was longer in lower latitude regions and shorter in higher latitude regions. Differences in the caudal appendage length among *O. albistylum speciosum* populations were explained by latitude rather than rearing temperature (Figure S1), implying a genetic origin. The caudal appendages are one of the indicators of defensive ability in dragonfly nymphs (Anisoptera).^39^ Our findings suggest that higher biodiversity and stronger defensive abilities in lower latitude regions may reduce the impacts of neonative species. However, it has also been reported that nymphs of Libellulidae with dorsal and lateral spines can be more vulnerable to invertebrate predators, such as nymphs of Aeshnidae, as these spines act as grips for the hooks and palpus of their labium, potentially lowering survival rates.^40^^,^^41^ The nymphs of *O. albistylum speciosum* lack dorsal and lateral spines and possess only caudal appendages, the functional role of which in interspecific competition remains unclear. Therefore, further investigation is needed to determine whether the observed patterns are directly influenced by the length of the caudal appendages.

Our results also showed that larger individuals of *T. aurora* had higher foraging intake (Table 2), suggesting that exploitation competition between the two species might intensify as they grow. Additionally, in Nara, the highest foraging intake of *T. aurora* was observed under the condition of 31°C (4°C above the current average temperature), which is the same as the warmest month average temperature in its native region, Taiwan.^20^ This suggests that the foraging intake of neonative species might increase in environments similar to its regions of origin. In contrast, no temperature effects were observed in other regions than Nara (Table 2). Since the collection site for *T. aurora* individuals used in all experiments was Kochi, genetic differences are unlikely. However, the foraging intakes of *O. albistylum speciosum* regional populations did not change under conditions of different temperature in the Hokkaido and Miyagi locations (Figures 2A and 2C), nor in Kochi under high temperatures (Figure 2E). Therefore, it should be noted that while interference competition with *T. aurora* was not detected in Kochi, a low-latitudinal region, exploitation competition might still occur.

Given the invasion status of *T. aurora* in Japan in 2024 (Figure 1) no clear influence of *T. aurora* toward *O. albistylum speciosum* could be found under the current average temperatures. It seems that the threat of the neonative dragonfly species on native species within the invaded regions was low even if *T. aurora* was present. On the other hand, the threat of *T. aurora* on native populations of *O. albistylum speciosum* may become clearer if the expansion of the invasive species to the north progresses further as the temperature rises.

### Conclusion

Our results support the hypothesis that there are the regional differences and unilateral ecological impacts by *T. aurora* (neonative) on *O*. *albistylum speciosum* (native), indicating that the impacts would be stronger in populations from higher latitudes than in those from lower latitudes (Figure 2; Table 1). Consequently, our findings highlight that even if there are no clear impacts by *T. aurora* at the present moment, they will definitely occur in the future if the expansion of this species to the north of Japan continues. Besides, it is an undeniable fact that no one has the power to stop the northward invasion of the neonative species as long as global temperatures keep rising. This valuable knowledge was obtained by comparing the ecological impacts of a neonative dragonfly species on its native counterpart among regions with different latitudes. Nevertheless, it is necessary to closely monitor the patterns of range expansion of neonative species and the potential impacts of long-term coexistence between the invader and native species, because the effect of time since *T. aurora*’s invasion was not sufficiently considered in this study. This highlights the importance of conducting an integrated monitoring at a nationwide and global scale for accurately assessing the threats of biological invaders associated with the global warming.

Our study using two dragonfly species with similar ecological niches demonstrated the following: (1) the impact of the poleward-expanding species on native species intensifies with the progression of warming even within the same location; and (2) the impact of the invader species is stronger in newly invaded environments, especially in high-latitudinal regions where the native species are less defensive against the newcomer. It is necessary to verify whether these results can be generalized to other organisms. Unlike the invasive exotic species resulting from human-mediated movement, the expansion of species associated with warming (neonative species) consistently exhibit a directional poleward invasion pattern whereby the effects of the latitude and temperature cannot be ignored.

### Limitations of the study

Our primary objective was to quantify the latitudinal gradient of the effects of invasive competitors attributed to warming on the food acquisition performance of native species, not to compare the relative strength of its inter- and intra-specific competitive effects. Nonetheless, there is no doubt that to properly predict the outcomes of competitive coexistence (or displacement), both intra- and inter-specific interaction parameters are needed. In this study, we examined only *O. albistylum speciosum* and *T. aurora*. However, in natural environments, other native species coexist in these habitats. The invasion of neonative species may further intensify competition among native species, highlighting the need for further research into these interactions. Additionally, our study focuses solely on interference competition, so we designed the experiments to include sufficient amounts of food, thereby preventing the occurrence of exploitation competition (indirect interspecific competition for resources). However, in real ecosystems, both interference and exploitation competitions can occur. This implies that the results of this study may underestimate the impact of *T. aurora* on *O. albistylum speciosum*.

## Resource availability

### Lead contact

Requests for further information and resources should be directed to and will be fulfilled by the lead contact, Masayoshi K. Hiraiwa (masayoshi.hiraiwa@gmail.com).

### Materials availability

This study did not generate new unique reagents.

### Data and code availability

• Previously published data were used for this work.^20^ The dataset for analyses is available at Figshare: https://doi.org/10.6084/m9.figshare.26519242.
• All original code used to perform analyses and generate figures is available at Figshare: https://doi.org/10.6084/m9.figshare.26519242.
• Any additional information required to reanalyze the data reported in this paper is available from the lead contact upon request.

## Acknowledgments

We would like to thank Dr. Takuo Sawahata, Dr. Jean B. Tanangonan, Dr. Mitsuo Matsumoto, Dr. Koya Hashimoto, and Dr. Yugo Seko for valuable technical advice. The authors thank Kazutaka Osaki, Shunsuke Yamamoto, Mitsuki Takano, and Ryota Shinotani for helping with our field sampling. We also thank Dr. Taku Kadoya and Dr. Koya Hashimoto for providing valuable information on sampling locations. We also appreciate Shunsuke Yamamoto for providing the photo of *T. aurora.* This work was supported by JSPS KAKENHI (grant number 19H03003 and 21K18318) and the Yakushima Environmental Culture Foundation.

## Author contributions

K.N.: conceptualization; data curation; formal analysis; investigation; methodology; project administration; resources; software; validation; visualization; writing—original draft; writing—review and editing. M.K.H.: conceptualization; data curation; formal analysis; investigation; methodology; software; validation; visualization; writing—original draft; writing—review and editing. N.I.: conceptualization; investigation; methodology. F.S-B.: writing—review and editing. D.H.: conceptualization; funding acquisition; methodology; project administration; resources; supervision; visualization; writing—original draft; writing—review and editing.

## Declaration of interests

The authors declare no competing interests.

## STAR★Methods

### Key resources table

**Table:** 

REAGENT or RESOURCE	SOURCE	IDENTIFIER
**Biological samples**		

*Trithemis aurora*	Field collection, Japan	N/A
*Orthetrum albistylum speciosum*	Field collection, Japan	N/A

**Deposited data**		

foraging test data	Nagano et al., 2023	https://doi.org/10.1098/rsos.230449

**Software and algorithms**		

R (ver. 4.1.3)	R Foundation	https://www.r-project.org/
Dataset	Figshare	https://doi.org/10.6084/m9.figshare.26519242
Code	Figshare	https://doi.org/10.6084/m9.figshare.26519242

### Experimental model and study participant details

#### Test species (*Trithemis aurora*, *Orthethrum albistylum speciosum*) and their ecological characteristics

As mentioned above, *T*. *aurora* was first discovered in Japan in the 1980s and has continued to move northward since then (Figure 1). The northernmost established population of *T. aurora* recorded to date is in Tokushima Prefecture.^28^ This neonative species has also been observed in Nara Prefecture^20^ but its establishment status is uncertain (Figure 1). *Trithemis aurora* is likely to continue expanding northward as global warming progresses. The average temperatures of the warmest month during the activity period of *T. aurora* nymphs in Nara, the northernmost invaded region at present, is 27°C based on the meteorological data from the past two decades (https://www.data.jma.go.jp/obd/stats/etrn/index.php). According to the global warming scenarios set by the Intergovernmental Panel on Climate Change (IPCC) AR6,^1^ temperatures are predicted to rise by +2°C under the SSP1-2.6 scenario and by +4°C under the SSP5-8.5 scenario during this century. Based on this prediction, the distribution of *T. aurora* is expected to spread throughout regions where the current mean temperature of the warmest month ranges from 23°C to 25°C by the end of 21th century and subsequently, would encounter native species dragonfly populations, including *O. albistylum speciosum* populations.

These two dragonflies share similar ecological characteristics, particularly at the nymphal stage.^20^ They can frequently be collected at the same site due to their inhabitations among the vegetation and surface layer of muddy soils, such as in rice paddies and ponds.^34^^,^^42^ Also, *O. albistylum speciosum* and *T. aurora*, which have a similar body size and life cycle period,^39^^,^^43^ will tend to forage on similar preys due to both having a similar shape at the tip of the lower lip.^32^^,^^44^ These characteristics suggest an intense interspecific competition (e.g., interference competition) for food acquisition between the two species. Some researchers indicate that resource competitions among Odonata nymphs can indirectly have an impact on their foraging intakes and mortalities.^21^^,^^45^^,^^46^^,^^47^

### Method details

#### Sampling and rearing of the two Odonata species tested

This study aimed to evaluate the differences in interspecies relationships between *T. aurora* and *O. albistylum speciosum* according to the latitudinal gradient of Japan, in order to assess the current and future risks posed by biological invasions of the poleward-expanding species (*T. aurora*) on native dragonflies (i.e., ecological threats under warming and/or bioinvasion scenarios). For this purpose, four *O. albistylum speciosum* populations from latitudes differing in the invasion status of *T. aurora* were selected: Kochi, a region where *T. aurora* already established around the 2000s; Nara, the northernmost region immediately after the *T. aurora* invasion^20^; Miyagi and Hokkaido regions, where the invasive species has not arrived yet but is expected to arrive during this century if the current warming trend continues unabated (Figure 1). The average temperature of the warmest month during the nymphal activity period in Miyagi (currently 25°C) is expected to reach 27°C by 2100 if the SSP1-2.6 scenario (+2°C) becomes a reality. Similarly, the temperature in Hokkaido (currently 23°C) is predicted to reach 27°C by 2100 under the SSP5-8.5 scenario (+4°C). This was the average temperature in Nara when *T. aurora* invaded the area in 2020.

In order to establish the reference population of the neonative species when conducting the comparative study, 10 adult females of *T. aurora* were collected from the pond in Aki City, Kochi Prefecture (33°29′32″ N, 133°56′30″ E) on 21 July 2023. This was done to ensure that all neonative dragonflies used in our experiments came from a synchronous source. Incidentally, the neonative species used in our previous study was also sampled from an area close to this collection site.^20^ As for the native dragonfly species, adult females of *O. albistylum speciosum* populations were collected from different latitudinal locations across the Japanese geography (Figure 1): a few females from the Nara population (34°40′22″ N, 135°43′47″ E) as described in our previous study^20^; five females from Kochi, were collected on the same day and pond as for *T. aurora*; 10 females were collected from Sendai City on 24-26 July 2023, Miyagi Prefecture (Tohoku district) (38°12′54″ N, 140°54′07″ E); and 5 females were collected from Sapporo City On 2-3 August 2023, Hokkaido (43°04′33″ N, 141°27′53″ E). After collection in each region, the tip of the abdomen of the dragonflies was inserted in a centrifuge tube filled with dechlorinated tap water to induce the deposition of eggs.

Breeding methods for both dragonflies were slightly modified from Nagano et al.^20^ Eggs of *T. aurora* from Kochi and three *O. albistylum speciosum* regional populations, namely Kochi, Miyagi and Hokkaido, were reared separately in plastic cups (66 mm diameter × 36 mm depth) containing 40 mL of dechlorinated tap water. Both eggs and their hatched nymphs were kept in a thermostatic incubator (LH-30-8CT/TG-180-3L/TG-180WLED-3LE/LP-30LED-8CTAR, Nippon Medical & Chemical Instruments Co, Ltd., Osaka, Japan). The time to hatch from the eggs was around 8 days irrespective of species or regional populations. Nymphs of both species were fed more nauplius larvae of *Artemia salina* L. (tetra brine shrimp, Spectrum Brands Japan, Inc. Kanagawa, Japan) than they could eat (saturating amount) once every second day during the rearing period.

Nymphs of *O. albistylum speciosum* regional populations were kept at a gradient of three temperatures tested in separate treatments each: 28°C, 30°C, 32°C for Kochi; 27°C, 29°C, 31°C for Nara; 25°C, 27°C, 29°C for Miyagi; and 23°C, 25°C, 27°C for Hokkaido. The lowest rearing temperatures (28°C Kochi, 27°C Nara, 25°C Miyagi, 23°C Hokkaido) represent the control in each population, as they match the average temperatures of the warmest month during the activity period of the nymphs of this species for the past two decades (https://www.data.jma.go.jp/obd/stats/etrn/index.php). As competition is expected to be more intense during the active season, we focused on temperature effects during this period rather than on the overwintering period, when activity levels are significantly reduced. The other two rearing temperatures indicate the average temperatures for warmest month predicted for increases of +2°C (SSP1-2.6) and +4°C (SSP5-8.5),^1^ compared respectively to the control (i.e., global warming scenarios). Nymphs of *Trithemis aurora* were reared at the same temperatures as the three *O. albistylum speciosum* regional populations. However, all *T. aurora* nymphs kept at 32°C died during the rearing period and thus, experiments for the native *O. albistylum speciosum* Kochi population were conducted only at two treatments:28°C (control) and 30°C (+2°C). Incubator photoperiod was set at 14:10 h (L:D) with reference to the sunlight hours of the warmest month at the nymphal activity period in each collection site of *O. albistylum speciosum*, and this setting was based on data from the National Astronomical Observatory of Japan (https://eco.mtk.nao.ac.jp/koyomi/dni/2021/s3008.html).

#### Foraging tests

In order to clarify the influence of the neonative species on the foraging behavior of the native species two separate experiments were conducted: tests under the pre-invasion and post-invasion scenarios of *T. aurora*. The experiment of the pre-invasion scenario allows to measure the foraging intake of prey by native species (*O. albistylum speciosum*) without a neonative species (*T. aurora*). The post-invasion test is almost identical to the pre-invasion scenario except that both species were present in the testing chamber. The methods of Nagano et al.^20^ with slight modifications were followed for these experiments.

Experimental procedures of the pre-invasion scenario of the neonative species for evaluating the foraging intake of nymphs of native dragonfly are as follows: (i) 200 nauplius larvae of *A. salina* were introduced into glass beakers (42 mm diameter × 61 mm depth) containing 40 mL of dechlorinated tap water and were held for 10 min for acclimation; (ii) for each temperature, 10 individuals (replicates) of native dragonfly species fasted 24-h were randomly selected for testing, and then contained one individual per beaker; (iii) foraging behavior of the nymphs of the native species against *A. salina* were recorded for an hour (the length of the experiment) using the rear camera of iPhone 7, iPhone 12, and iPod touch (Apple Inc. USA), and subsequently counted the number of the prey animals remaining to estimate the foraging intake (i.e., the number of prey consumed). For the post-invasion scenario test, a pair of nymphs of the two dragonfly species were randomly selected and put together in a beaker; the foraging intake of native species in the presence of a neonative species was counted following the same procedure as in the pre-invasion test. Thereafter, results for both scenarios were compared for each temperature treatment. To remove effects of individual differences, the post-invasion test utilized the same individuals as those used in the pre-invasion scenario. However, if any of the tested individuals were preyed by a neonative species, the corresponding data were excluded from the analyses. Given the effects of the date tested against these results, multiple dates were set for experiments and individuals, so that the test of the pre-invasion scenario was first conducted and the post-invasion test was divided by half (5 individuals) for each population. Foraging experiments using nymphs about two-months old (i.e., after hatching) were conducted for four *O. albistylum speciosum* regional populations including Nara population^20^ and *T. aurora*. Also, the tests were only conducted during the daytime when the light in an incubator turned on (i.e., 9.00 to 20.00) for all pairs and treatments to remove the influence of the testing time and photoperiod on the foraging behavior of each species/population. The length from the head without antenna to the abdomen including caudal appendages of each test individual was measured before the experiments^20^ for assessing the effect of the body size on the interference competition between the two dragonfly species.

After that, to validate whether the neonative species might also be more vulnerable than the native species, we complementary conducted the foraging test under the same temperature conditions. The test focused exclusively on the foraging intake of the neonative species in the absence of native species and thus, were compared it with the foraging intake of *T. aurora* observed in the post-invasion scenario.

#### Latitudinal comparison of morphological traits of *O. albistylum speciosum*

To evaluate the latitudinal gradient in the defensive ability of the native species from competitors or predators, we measured the length of the caudal appendages of all *O. albistylum speciosum* nymphs tested as this is an indicator of the defensive ability of dragonfly nymphs.^39^ For a broader, nationwide assessment of the impacts of *T. aurora* on *O. albistylum speciosum*, females of the latter species were also collected from Kagoshima and Ibaraki prefectures in addition to the four regions (Kochi, Nara, Miyagi, and Hokkaido prefectures) where the foraging tests were conducted (Figure 2).

On 30 June to 2 July 2022, 5 females were collected in Minamisatsuma City, Kagoshima Prefecture (southern Kyushu district) (31°26′26″ N, 130°17′21″ E). On 20-21 July 2022, 7 females were sampled from Tsukuba City, Ibaraki Prefecture (northern Kanto district) (36°02′57″ N, 140°07′09″ E). Also, on 26-29 June 2022, we tried to sample *O. albistylum speciosum* females in Okinawa Prefecture. Although Okinawa is within the distribution range of *O. albistylum speciosum*, its current population is quite low due to a sharp decline in rice paddies and wetlands (Okinawa Prefectural Government 2017) and thus, no females could be collected during the survey period—this region was, therefore, excluded from analysis. Eggs were collected from the captured females (see “foraging tests” for the method of producing eggs), and nymphs were reared from those eggs. After hatching, the length of the caudal appendages and body sizes of each regional population were measured.

### Quantification and statistical analysis

To evaluate whether the foraging intakes of each population of native species differ depending on the temperature rise (+2°C, +4°C from the controls) and/or bioinvasion (pre- or post-invasion of *T.aurora*) scenarios, we conducted generalized linear mixed models (GLMMs) with Poisson errors and log link. We conducted these analyses for each of the four *O. albistylum speciosum* populations, resulting in a total of four analyses (4 populations). The response variables were the intakes of either four *O. albistylum speciosum* populations including Nara population^20^ in two foraging tests, i.e., pre- and post-invasion scenarios of the neonative species. Incidentally, the analysis for the Nara population was reported on a previous study,^20^ and analyses for the other populations were conducted following the same methodology. The explanatory variables included the following four factors: temperature, *T. aurora* invasion scenarios, body size, and their interactions (temperature × invasion scenarios, body size × invasion scenarios). Individual differences in female adults for producing eggs and their nymphs tested were included as nested random effects, and testing dates were fitted as random effect. Given these, we firstly performed the analysis using the full model that included interactions, and then if the interactions were not significant, we reanalyzed the data excluding the non-significant interactions.

Similarly, to evaluate whether the foraging intakes of the neonative species differ depending on the temperature rise and/or presence/absence of a native species, we applied the same GLMM framework with Poisson errors and log link. The explanatory variables in this analysis included temperature, presence or absence of a native species, body size, and their interactions (temperature × native species, body size × native species). As with the native species, random effects included individual differences and testing dates, and the same analytical procedures were applied.

To examine whether the ratio of the caudal appendage length to body size in *O. albistylum speciosum* nymphs varies with latitude, we fitted the data using GLMM. The response variable in this case was the ratio of caudal appendage length to body size, with inhabitation latitude and rearing (treatment) temperature in each regional population as explanatory variables, and sampling sites as a random effect. Incidentally, multi-collinearity was not detected between the latitude and temperature (Variance Inflation Factor = 1.584).

All statistical details, including estimates, standard errors (S.E.), z values, and *p* values, are reported in Tables 1 and 2. The fitted curves are presented with 95% confidence intervals, as shown in Figure 2. Due to variation across sites, temperature conditions, and invasion scenarios, sample sizes (n) varied among treatments. Full details of the sample sizes for each experimental condition are provided in the deposited dataset (https://doi.org/10.6084/m9.figshare.26519242). Statistical analyses were conducted using R ver.4.1.3.^48^ The GLMMs were conducted using the package “glmmTMB”.^49^
