# Supplementary material for: Different response of a native dragonfly species against a neonative invader along a latitudinal gradient
Source: iScience. 2025 Jun 13;28(7):112887. doi: 10.1016/j.isci.2025.112887 (PMC12272760; doi:10.1016/j.isci.2025.112887)
Supplement: Document S1. Figure S1 [file mmc1.pdf]

iScience, Volume *28*

Supplemental information

Different response of a native dragonfly species against a neonative invader along a latitudinal gradient

Koki Nagano, Masayoshi K. Hiraiwa, Naoto Ishiwaka, Francisco Sánchez-Bayo, and Daisuke Hayasaka

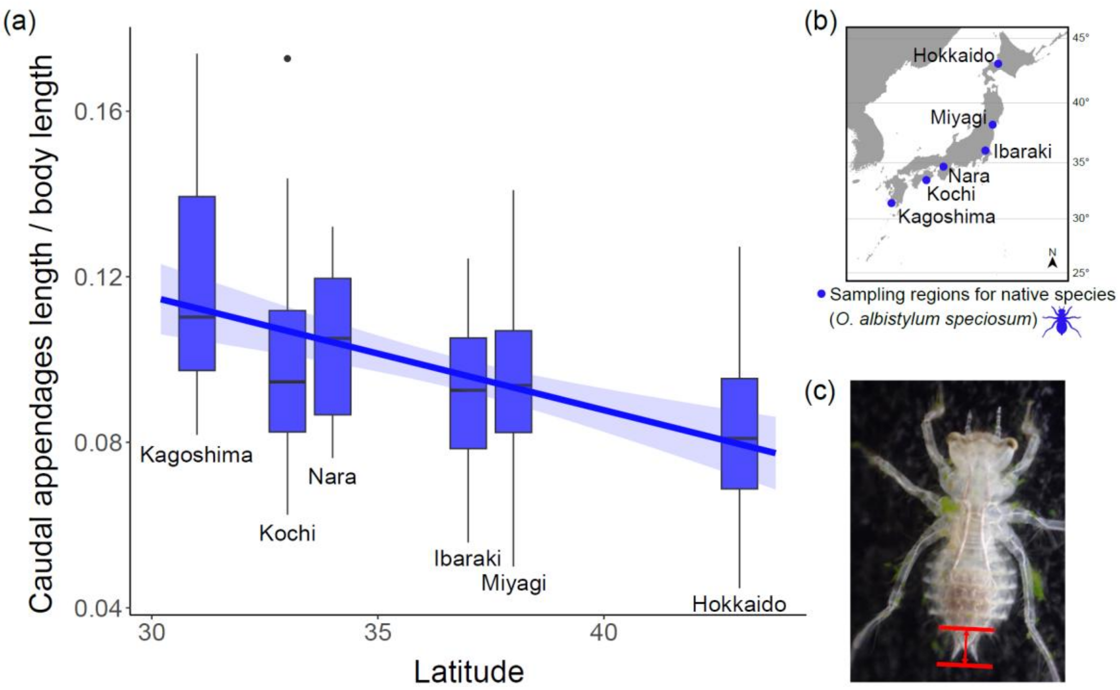

Figure S1. Latitudinal gradient in relative caudal appendage length in *O. albistylum speciosum* nymphs

(a) Latitudinal trend in the ratio of the length of the caudal appendages (mm) to body size (mm) among six *O. albistylum speciosum* regional populations. Fitted curves and bands for 95% confidence interval with GLMM. (b) Map showing the sampling regions for *O. albistylum speciosum* across different latitudes in Japan. (c) Bar in red shows the position of the caudal appendages of odonata nymphs (*O. albistylum speciosum*).
